# MiR-15a Decreases Bovine Mammary Epithelial Cell Viability and Lactation and Regulates Growth Hormone Receptor Expression

**DOI:** 10.3390/molecules171012037

**Published:** 2012-10-12

**Authors:** Hui-Ming Li, Chun-Mei Wang, Qing-Zhang Li, Xue-Jun Gao

**Affiliations:** Key Laboratory of Dairy Science of Education Ministry, Northeast Agricultural University, Harbin 150030, Heilongjiang, China; Email: swx03390lhm@126.com (H.-M.L.); wangcm-1@sohu.com (C.-M.W.); gaoxj5390@sina.com (X.-J.G.)

**Keywords:** bta-miR-15a, *Ghr*, proliferation, bovine mammary epithelial cell

## Abstract

MicroRNAs (miRNAs) are a class of small non-coding RNAs that regulate the expression of target genes at the post-transcriptional level by transcript degradation or translational inhibition. The role of bta-miR-15a in bovine mammary gland hasn’t been reported. Using miRNAs prediction software, GHR gene was predicted to be a potential target of bta-miR-15a. In this study, bovine mammary epithelial cell line was used as an *in vitro* cell model to address the function of bta-miR-15a on bovine mammary epithelial cells. The expression changes of bta-miR-15a and *Ghr* after bta-miR-15a transfection were detected by qRT-PCR; the expression of GHR protein and casein was detected by western blotting. To determine whether bta-miR-15a can affect cell viability, cells were examined using an electronic Coulter counter (CASY-TT). In conclusion, bta-miR-15a inhibited the expression of casein of bovine mammary epithelial cells, and cell number and viability were reduced by bta-miR-15a expression. Bta-miR-15a inhibited the viability of mammary epithelial cells as well as the expression of GHR mRNA and protein level, therefore suggesting that bta-miR-15a may play an important role in mammary gland physiology.

## 1. Introduction

MicroRNAs (miRNAs) are located within introns of coding or noncoding genes, exons of noncoding genes or in intergenic regions [1]. They are small, noncoding RNAs that regulate gene expression by catalyzing the cleavage of messenger RNA (mRNA) [2] or repressing mRNA translation [3,4,5]. They are involved in the regulation of many important physiological processes [6,7], such as the regulation of cell proliferation and metabolism [8], developmental timing [9,10], cell death. Some studies showed that miRNAs were involved in the regulation of mammary gland [11,12], the organ that can undergo cycles of cell division, differentiation and dedifferentiation in the adult, but the details of miRNAs on bovine mammary gland was poorly understood. 

Bovine miR-15a (bta-miR-15a) is located in bovine chromosome 12 between 18887743bp and 18887825bp [13], It was verified to be critical in cell development [14], cell cycle [15] and death [16,17]. MiRNAs have been reported regulate the mammary gland development [11]. And qRT-PCR can detect the expression of miR-15a in mammary gland. So it was proposed that bta-miR-15a could regulate the mammary gland development of Holstein dairy cows. 

Growth hormone (GH) is one of the critical regulators of growth and metabolism [18]. It can increase milk production in cattle [19]. GH interacts with GH receptor (GHR), a cytokine superfamily receptor, to activate the cytoplasmic tyrosine kinase, Janus kinase2, and initiate intracellular signaling cascades. GH receptor is non-kinase recepors whose activation of signaling pathways requires participation of receptor-associated kinases, such as Janus kinases or Src kinases. Signal transduction by the recepors mainly involves the JAK/Stat pathway [20]. GHR is a transmembrane receptor for growth hormone encoded by the GHR gene [21]. Binding of GH to its receptor leads to receptor dimerization and the activation of intercellular signal transduction pathway leading to growth [22]. Recent findings that GH receptor (GHR) mRNA and protein are expressed in the epithelial cells of the bovine mammary gland suggest that GH may directly act on these cells to affect milk production. Indeed GHR is found to be associated with milk yield and composition and can indirectly regulate milk production [23]. GHR gene was associated with a strong effect on milk yield and composition. The growth hormone receptor is a transducer for growth hormone action, which plays a pivotal role in the lipid and carbohydrate metabolism. Furthermore, it has a major role in the growth hormone axis through the initiation and maintenance of lactation. GHR gene mutation showed strong effects on milk protein [24]. Using TargetScan5.1 predict software, GHR gene was predicted to be the potential target gene of bta-miR-15a. So it is necessary to confirm the regulatory relationship between bta-miR-15a and GHR. In this study, bta-miR-15a mimics or inhibitor were transfected into bovine mammary epithelial cells respectively. Using quantitative real-time PCR, Western blotting, and CASY TT techniques the expression of lactation related genes and proteins was addressed and analyzed. 

## 2. Results and Discussion

### 2.1. Bta-miR-15a Was Transfected Efficiently

**Figure 1:**
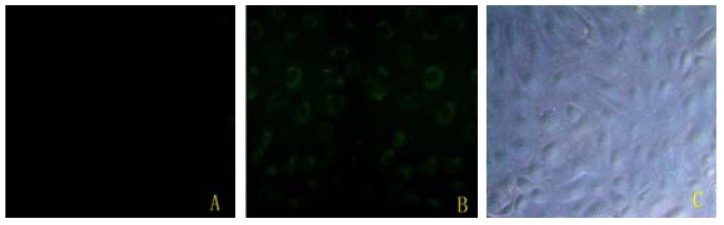
Bta-miR-15a mimics-FAM transfect into bovine mammary gland epithelial cells. (**A**) Non-transfected mammary gland epithelial cells, under fluorescent microscope (100×); (**B**) Mammary gland epithelial cells transfected with bta-miR-15a mimic-FAM, under fluorescent microscope (100×); (**C**) Mammary gland epithelial cells transfected with bta-miR-15a mimic-FAM, under light microscope (100×).

The result in Figure 1 showed that bta-miR-15a was successfully transfected into BMECs. Using fluorescent microscope, green fluorescence labeling was detected in the cells that were transfected bta-miR-15a mimics-FAM. Compared to non-treated group cells, there was no green fluorescence labeling detected. The efficiency of transfection is the amount of have green fluorescence label cells divides by the total amount of the cells. So the transfection efficiency is about 70–80% in Figure 1.

### 2.2. Ghr Were Predicted to Be the Target of Bta-miR-15a

**Figure 2:**
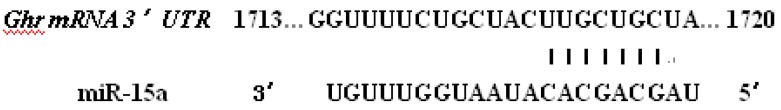
miR-15a targets 3'UTR of Ghr mRNA.

Using TargetScan5.1 predict software, *Ghr* was predicted to be the potential target gene of bta-miR-15a (Figure 2):

### 2.3. Expression of Ghr mRNA in Bta-miR-15a Mimics/Inhibitor Transfected BMECs

**Figure 3:**
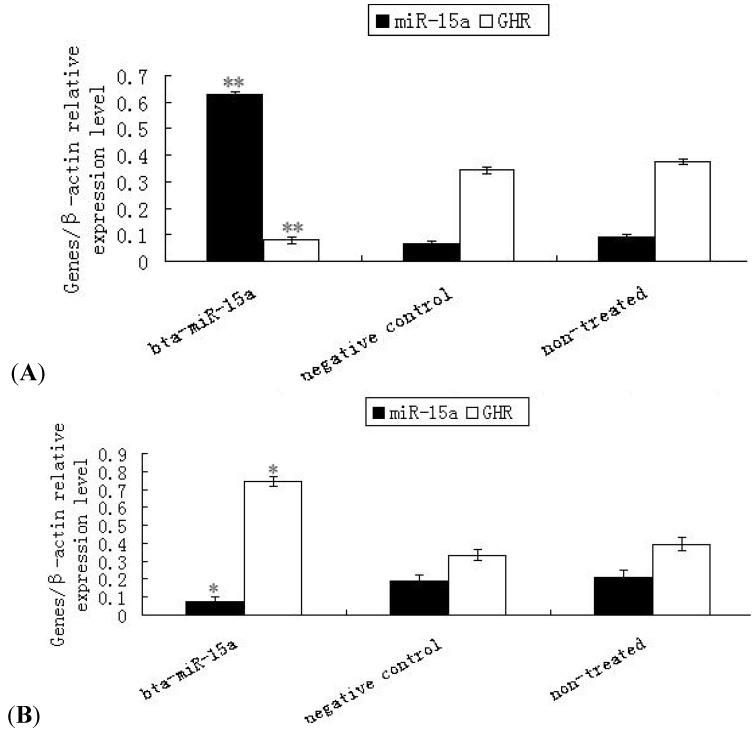
Expression of bta-miR-15a and *Ghr* mRNA detected by quantitative real-time PCR in bovine mammary epithelial cells transfected with bta-miR-15a mimics (**A**) or bta-miR-15a inhibitor (**B**). (**A**) An Endogenous miR-15a was raised in the cells with overexpress-miR-15a. ** *p* < 0.01. Expression of *Ghr* mRNA in bovine mammary epithelial cells after miR-15a mimics transfect was decreased. ** *p* < 0.01. (**B**) Endogenous miR-15a was decressed and expression of *Ghr* mRNA was raised after miR-15a inhibitor transfect. * *p* < 0.05. Values are means ± SEM (n = 3 per group). * and ** indicate significant differences from values obtained in the control group at levels of *p* < 0.05 and *p* < 0.01, respectively.

To investigate the potential regulatory role of bta-miR-15a on *Ghr* mRNA, we examined the mRNA expression of bta-miR-15a and *Ghr*. The results showed that when bta-miR-15a was over-expressed by transfection, the expression of the bta-miR-15a was 8.4 fold higher relative to the endogenous level and the expression of *Ghr* decreased (*p* < 0.01) (Figure 3A). Figure 3B showed that when bta-miR-15a was inhibited by transfection, the expression of the bta-miR-15a was lower than the endogenous level and the expression of *Ghr* increased (*p* < 0.05). The results revealed that bta-miR-15a could inhibit *Ghr* transcription in mammary epithelial cells

### 2.4. Expression of GHR Protein and Casein Synthesis in Bta-miR-15a Mimics/Inhibitor Transfected BMECs

**Figure 4:**
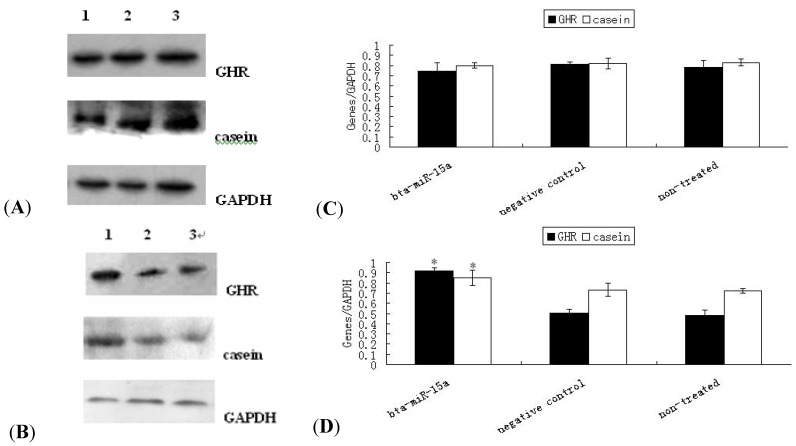
Protein grayscale scanning and statistical results of GHR and casein by Western blotting. (**A**) Immunoblot of GHR and cansein in mammary epithelialcells the 2 days after transfect with miR-15a mimics, negative control, and non-transfected group. The mediated expression of miR-15a mimics down-regulated GHR protein and casein transcribed from the endogenous *Ghr* transcript, compared to control and non-transfected group. 1–3 indicate bta-miR-15a mimics group, negative control group and non-treated group respectively; (**B**) Immunoblot of GHR and cansein in mammary epithelialcells the 2 days after transfect with miR-15a inhibitor, negative control, and non-transfected group. The mediated expression of miR-15a inhibitor up-regulated GHR protein and casein transcribed from the endogenous *Ghr* transcript, compared to control and non-transfected group. 1–3 indicate bta-miR-15a inhibitor group, negative control group and non-treated group respectively; C, D respectively shows expression protein relative levels of GHR and casein by transfect bta-miR-15a mimics and bta-miR-15a inhibitor. Protein expression of GHR and casein normalize by GAPDH. Values are means ± SEM (n = 3 per group). * indicate significant differences from values obtained in the control group at levels of *p* < 0.05.

Western blotting results showed that the expression of GHR and casein were reduced by bta-miR-15a transfection. Figure 4A,C and Figure 4B,D showed that compare to the negative control group and non-treated group, the expression of GHR and casein were both decreased (*p* > 0.05) and increased (*p* < 0.05) in transfected bta-miR-15a transfection groups respectively. So the results suggested that bta-miR-15a could also inhibit the expression of GHR protein at translational level and affected the synthesis of casein indirectly, but the mechanisms were still unknown.

### 2.5. Bta-miR-15a Reduced Cell Viability

The CASY-TT Analyser System can determine the cell number and size distribution in a sample quickly and reliably. The viability of the cells is measured directly. The aggregation level of the cells is determined and automatically included in the calculation of the cell concentration. In this study, same amount of cells were seeded on cell culture plates as previously described. The cells were harvested at 24 h post-transfection of bta-miR-15a (Table 1). Cell viability was recorded as percentage of viable cells in total cells. CASY-TT measured that the cell viability of bta-miR-15a mimics/inhibitor transfected group relative to the negative control group was reduced 17.66% and increased 4.28%, respectively (*p* < 0.01, *p* < 0.01 respectively). The results revealed that bta-miR-15a could inhibit cell proliferation and depress cell viability.

**Table 1:** Result of bovine mammary epithelial cells viability. (**A**) Over-expressed bta-miR-15a. (**B**) Inhibited bta-miR-15a. The data were analyzed with Sigmaplot 9.0. Values are means ± SEM (n = 3 per group). Significantly difference between a and b, *p* < 0.01.

**A.** Effect of bta-miR-15a mimics transfection on BMECs viability.	
**Group**	**Viability (%)**
miR-15a mimics 30 nM	64.69 ± 0.60 ^b^
Negative control	83.52 ± 1.26 ^a^
Non-transfected group	82.35 ± 0.80 ^a^
**B.** Effect of bta-miR-15a inhibitor transfection on BMECs viability	
**Group**	**Viability (%)**
miR-15a inhibitor 30nM	89.43 ± 0.18 ^b^
Negative control	82.78 ± 0.62 ^a^
Non-transfected group	83.15 ± 0.50 ^a^

### 2.6. Discussion

MiRNAs are a class of small RNA that was associated with many biological functions. Although the specific biological roles of some miRNAs have been reported, information about the role of miRNA in bovine mammary glands is limited, so a study on miRNA in bovine mammary gland is necessary. In this work, the effects of bta-miR-15a on *Ghr* were investigated, and the role of bta-miR-15a in cultured bovine mammary epithelial cells was demonstrated.

The mammary gland represents unique tissue, which undergo cycles of cell proliferation, differentiation, and apoptosis during adult life. MicorRNAs have the function of regulating cell proliferation, differentiation, apoptosis, and other life processes, so the miRNAs may play an important effect on mammary gland. Previously it was reported that many miRNAs existed in mammary gland. It was reported that miR-101a controls mammary gland development by regulating cyclooxygenase-2 expression [25].

Mir-15a and miR-16-1 is located on human chromosome 13q14, in 68% of the B-CLL two gene expression level decreased [16]. Cimmino was further confirmed the expression were negatively correlated of miR-15a and miR-16-1 and BCL-2 expression in CLL cells.MiR-15a control of Bmi-1 gene expression in ovarian carcinoma [26]. In addition, miR-15a/miR-16 control cell cycle in non-small lung cancer [15], so since in different tissues miR-15a have different functions, we speculated that miR-15a can regulate mammary gland acting in a different role.

Mammary gland development is controlled by a complex interplay of endocrine hormones, in particular growth hormone, estrogen, progesterone, and prolactin, acting together.GHR as an important cytokine superfamily receptor involved in many signaling cascades drew a lot of attention. GHR also plays an important role in mammary glands, and it was found to be associated with mammary gland development, lactation and composition of milk [27]. Although there were many studies on GHR, the regulatory mechanisms involved were not fully investigated [28]. In this study, we found that the expression of GHR mRNA and protein was regulated by transfected bta-miR-15a synthetics. This work suggested a novel regulatory mechanism of GHR by miRNAs.

GH must bind GHR in the first step to trigger a signal transduction process. The downstream signaling pathways of GHR are induced by JAK2. Induced JAK2, the downstream signaling cascades include four ways: signal transduction and activator of transcription (STAT) pathway, mitogen-activated protein kinase (MAPK) pathway, protein kinase C (PKC) pathway and the insulin receptor substrate (IRS) approach. STAT plays an important role in the regulation of mammary gland development and milk protein synthesis. Stat5 gene is an important regulatory factor in milk protein synthesis. It was reported that there was at least one binding site on the β-casein promoter [29]. GHR acts through the STAT5 pathway to regulate the expression level of casein. MAPK can promote mammary epithelial cell proliferation and cell cycle. PKC plays an important role in glucose metabolism. IRS can maintain cell growth, division and metabolism. GHR is a very important receptor, so research on GHR is important to regulate cow mammary gland development, lactation and the quality of milk. Previous reports state that GH can activate β-casein in mammary explant cultures. The lactogenic activity of GH may be elicited directly through the activation of Stat5 followed by the activation of milk protein gene transcription in alveolar cells [30], so we suggest that the operative mechanism was that miR-15a deregulated GHR, affected JAK2-Stat5 signal pathway and decreased the expression of β-casein. 

It was known that the number and the viability of mammary epithelial cells are associated with milk production [31], so an increased number of mammary epithelial cells and enhanced cell viability will contribute to lactation, but the mechanisms that are responsible for the variations in the activity and number of mammary cells during lactation in ruminants are still poorly understood [32]. In this study, our results showed that over-expression of bta-miR-15a decreased the viability of mammary epithelial cells; on the contrary, the expression of bta-miR-15a inhibitor increased the viability of mammary epithelial cells, suggesting bta-miR-15a could regulate cell viability. In our work, the signal pathway by which the miR-15a regulated cell viability was not clearly identified. Here we suggest bta-miR-15a as a novel regulator to promote lactation in mammary epithelial cells of dairy cows.

In general, the gross composition of cow’s milk is 87.7% water, 4.9% lactose (carbohydrate), 3.4% fat, 3.3% protein, and 0.7% minerals (referred to as ash). In cow's milk, approximately 82% of milk protein is casein. Casein, a major milk protein, also revealing the ability of mammary epithelial cells, was down-regulated by miR-15a. This study showed that miR-15a regulates GHR, changes the viability of mammary epithelial cells and expression of casein. The expression of casein was decreased by miR-15a mimic transfection. This implied that miRNAs cannot be overlooked as a class of molecules regulating biological functions and mammary gland development. Our data enhance current understanding of the functions of miRNAs on mammary gland development and lactation.

## 3. Experimental

### 3.1. Cell Culture

Bovine mammary gland epithelial cells (BMECs) were provided by Key Laboratory of Dairy Science of Education Ministry (Northeast Agricultural University, Harbin, China). Dulbecco’s modified Eagle’s medium-F12 (Gibco) containing 10% heat inactivated FBS, 5 μg/mL insulin (Sigma, Oakville, ON, Canada),100 U/mL penicillin and 100 μg/mL streptomycin were used for routine maintenance of BMECs. For experimental assays, the logarithmic growth phase of mammary gland epithelial cells was cultured on glass cell culture bottles in a humidified atmosphere with 5% CO_2_ at 37 °C.

### 3.2. Transient Transfection

Logarithmic growth phase of mammary gland epithelial cells were plated at 6 × 10^4^ cells/well on 24 well cell culture plates and were separated in three groups. One group of cells was cultured with basal medium as normal control, called non-treated group, which for the other two groups was transfected with bta-miR-15a mimics/inhibitor and miR-15a negative control respectively. Three parallel groups were prepared for each treatment. Bta-miR-15a mimics/inhibitor and miR-15a negative control was transfected followed siPORT *NeoFX*^TM^ Transfection Agent manufacturer’s protocol (Ambion, Austin, TX, USA) and the final RNA concentration is 30 nM for bta-miR-15a mimics/inhibitor/negative. Transfect efficiency were detected by fluorescence microscope and harvested the cells at 24h after transfected.

### 3.3. Quantitative Real-Time PCR Analysis

Total RNA was extracted from all group cultured cells using Trizol reagent (Invitrogen, Carlsbad, CA, USA) according to the manufacturer’s protocol [33]. Total RNA concentration was determined by the measurement of optical density at 260 nm with an ultraviolet spectrophotometer (Beckman spectrophotometer; Beckman Coulter, Inc.; Fullerton, CA, USA). Total RNA integrity was verified by an OD260/OD280 absorption ratio greater than 1.8 [34]. From each sample preparation, 1µg of total RNA was used to synthesize first strand cDNA by reverse transcription with TaKaRa’s PrimerScript RT-PCR kit (TaKaRa, Tokyo, Japan) by following the manufacturer’s instructions.

RT-negative controls were included in each batch of reactions. Real-Time-PCR quantification of bta-miR-15a expression was performed using Hairpin-it^TM^ miRNAs qPCR Quantitation Kit (GenePharma, Shanghai, China) according to the manufacturer’s protocol. PCR reactions were carried out in final volumes of 20 μL using an ABI PRISM 7300 Real-Time PCR System (Applied Biosystems, Foster City, CA, USA). Briefly, reactions consisted of 2 × Real time PCR Buffer 10 μL; miR-specific Primer set 0.32 μL; miRNA RT product 2 μL; Taq DNA polymerase 0.2 μL; ddH_2_O 7.48 μL; Reactions were initiated with a 3-min incubation at 95 °C followed by 40 cycles of 95 °C for 12 s and 62 °C for 40 s. The specific primer of bta-miR-15a was designed by company of GenePharma. 

Real-Time-PCR analysis of *Ghr* and β-actin was performed under the following conditions: denaturation at 95 °C for 10 min, followed by 40 cycles (95 °C for 12 s, 62 °C for 40 s for GHR and β-*actin* genes, respectively). Sequences of primer pairs used were as follows: 5'-CGTGGACAACGCTTACT-3' and 5'-AAGGGTTTCTGTGGTGAT-3' for *Ghr* and 5'-TTAGCTGCGTTACACCCTT-3' and 5'-GTCACCTTCACCGTTCCA-3' for β*-actin*, the primers were synthesized by the company of Invitrogen.

Product purity was confirmed by melting curve analysis [35]. All real-time PCR assays were performed in duplicate in at least three independent experiments using three different treatments. The relative quantity of *Ghr* and bta-miR-15a gene expression was calculated using the comparative cycle threshold (ΔΔCt) method [36]. The expression level of the *Ghr* and bta-miR-15a gene was then normalized for each sample using β*-actin* as a reference gene.

### 3.4. Western Blotting

Three group culture cells were harvested at 24 h by transfected. The cells lysates were prepared and resolved by SDS-PAGE (20 μg protein/sample) as described [1,7]. Western blotting was performed with a polyclonal rabbit antibody to human GHR, casein and GAPDH (Santa Cruz Biotechnology, Santa Cruz, CA, USA), respectively. Goat-anti-rabbit antibodies conjugated to horseradish perxidase and the Super ECL plus (ApplyGEN, Beijing, China) were used to detect protein bands. Analysis of the expression of GHR and casein was using GAPDH as a reference protein. 

### 3.5. Quantification of Cell Viability

The numbers of adherent cells were determined with a Cell Counter Analyser System Model TT (CASY TT) Analyser System (Scharfe System GmbH, Reutlingen, Germany) was used according to the manufacturer’s instructions. After calibration with dead and vital mammary epithelial cells, cursor positions were set to 11.75 to 50.00 μm (evaluation cursor) and 7.25 to 50.00 μm (standardization cursor). Cells were trypsinized. The cells diluted with CASY electrolyte solution (1:100) was examined using the CASY-TT electronic Coulter counter; 100 μL aliquots were analyzed in triplicate [37].

### 3.6. Statistical Analysis

Results were reported as mean ± SEM. Statistical analysis was done using Sigmaplot 9.0 software. Gray-scale scanning of Western blotting results was analyzed by BandScan 4.3 software. The data were analyzed using Student’s *t* test, with a *p* value of <0.05 considered signiﬁcant.

## 4. Conclusions

The present study showed that bta-miR-15a negatively regulated the expression of *Ghr*, confirming the prediction by bioinformatics that the *Ghr* gene could be a potential target of bta-miR-15a. Bta-miR-15a could reduce the expression of GHR and casein proteins produced by bovine mammary epithelial cells. In addition, bta-miR-15a reduced the viability of mammary epithelial cells. Therefore we propose that expression of bta-miR-15a regulates bovine mammary gland development and lactation. These results could be a theoretical basis for future research on bovine mammary gland development and lactation biology.
